# Supplementary figures and images for: Shiny-SoSV: A web-based performance calculator for somatic structural variant detection
Source: PLoS One. 2020 Aug 27;15(8):e0238108. doi: 10.1371/journal.pone.0238108 (PMC7451576; doi:10.1371/journal.pone.0238108)

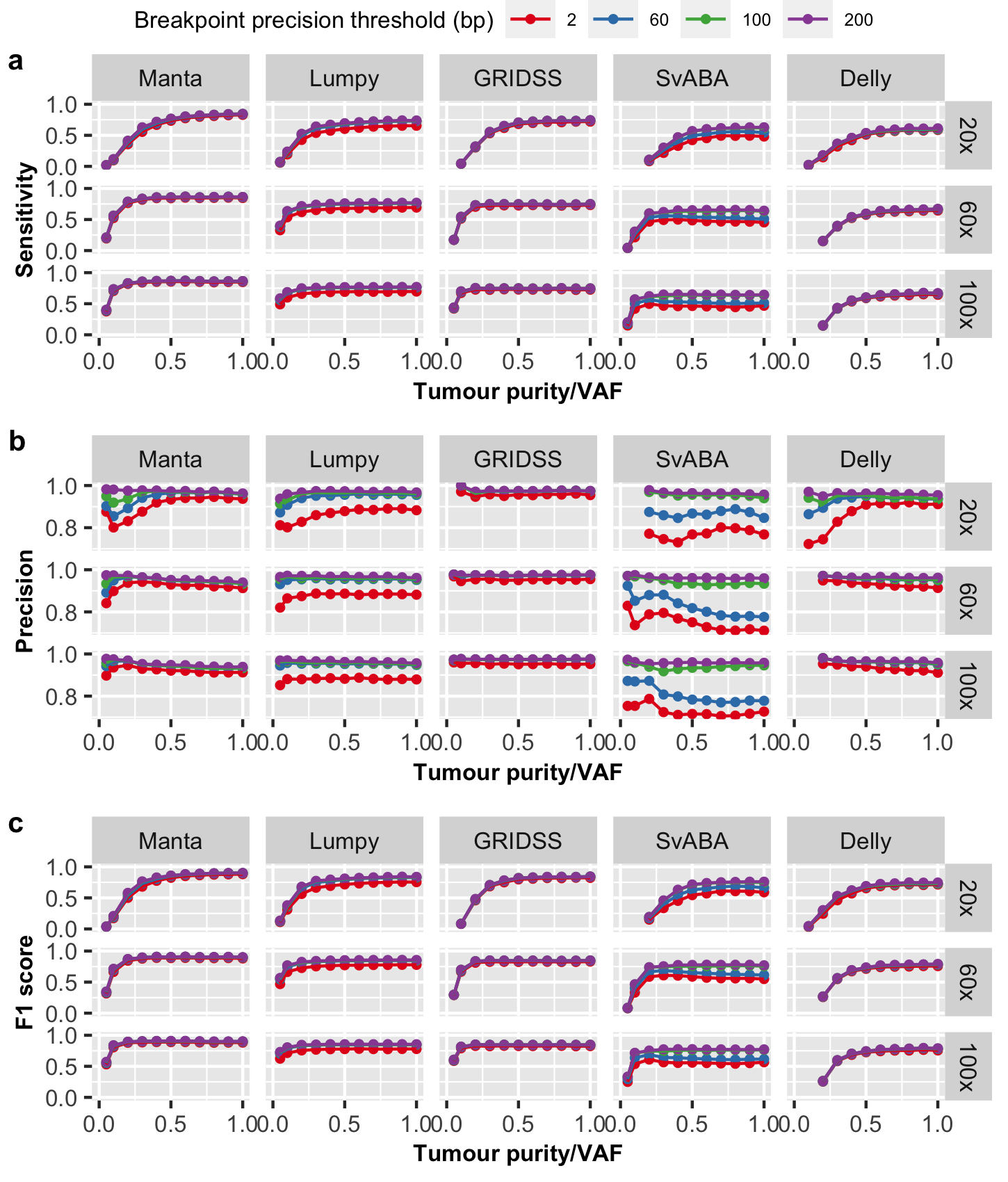

Supplement: S1 Fig — Shown are the effect of the interaction of VAF and breakpoint precision threshold (by colour) on sensitivity (a) and precision in log scale (b) for five SV callers (Manta, Lumpy, GRIDSS, SvABA, Delly). Results are based on simulation data set with tumour coverage of 20x, 60x and 90x, breakpoint precision threshold of 2bp, 60bp, 100bp and 200bp and normal coverage of 60x. (TIF) [file pone.0238108.s001.tif]

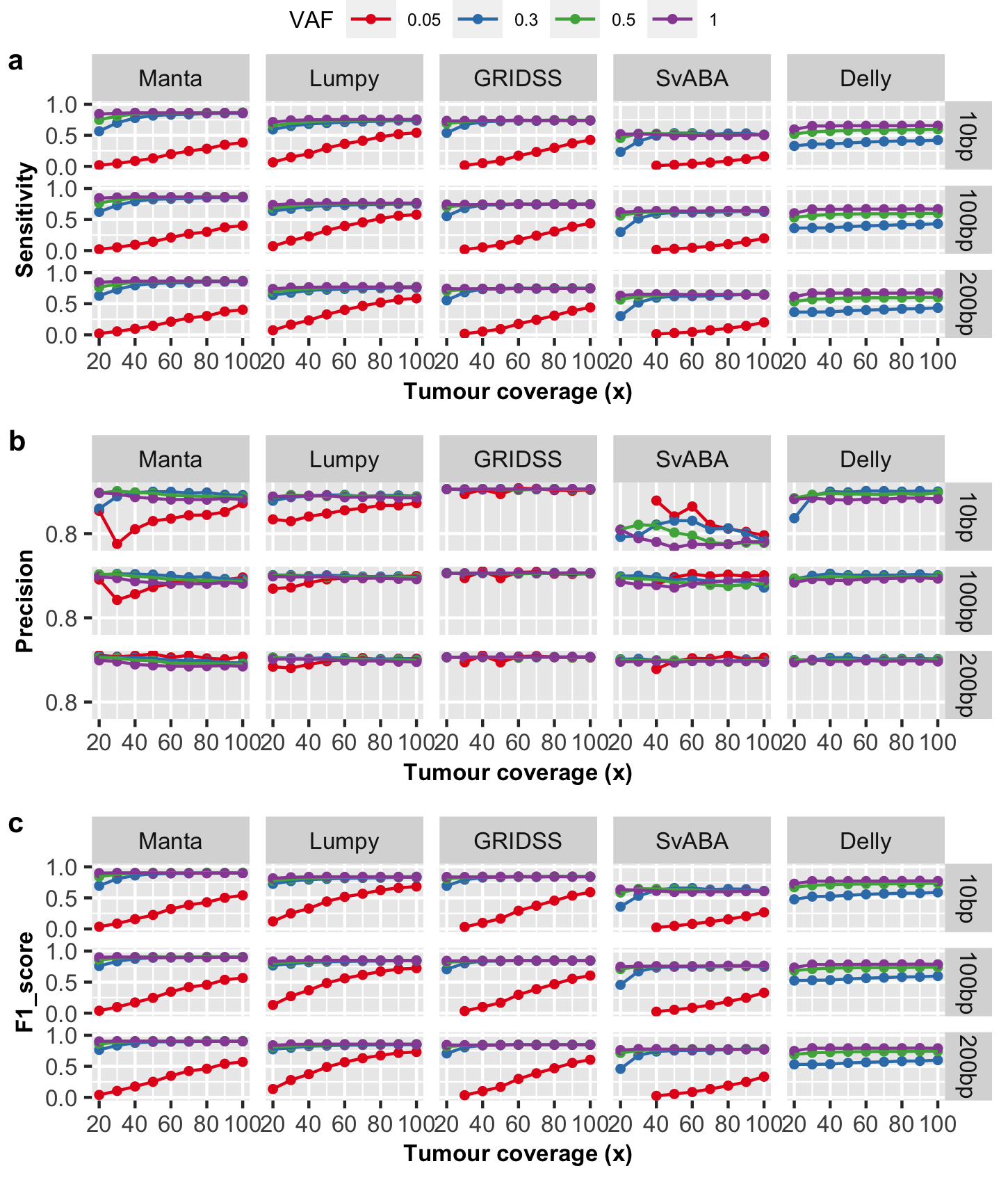

Supplement: S2 Fig — Shown are the effect of the interaction of tumour coverage and VAF (by colour) on sensitivity (a) and precision in log scale (b) for five SV callers (Manta, Lumpy, GRIDSS, SvABA, Delly). Results are based on simulation data set with breakpoint precision threshold of 10bp, 100bp and 200bp, VAF of 0.05, 0.3 0.5 and 1 and normal coverage of 60x. (TIF) [file pone.0238108.s002.tif]

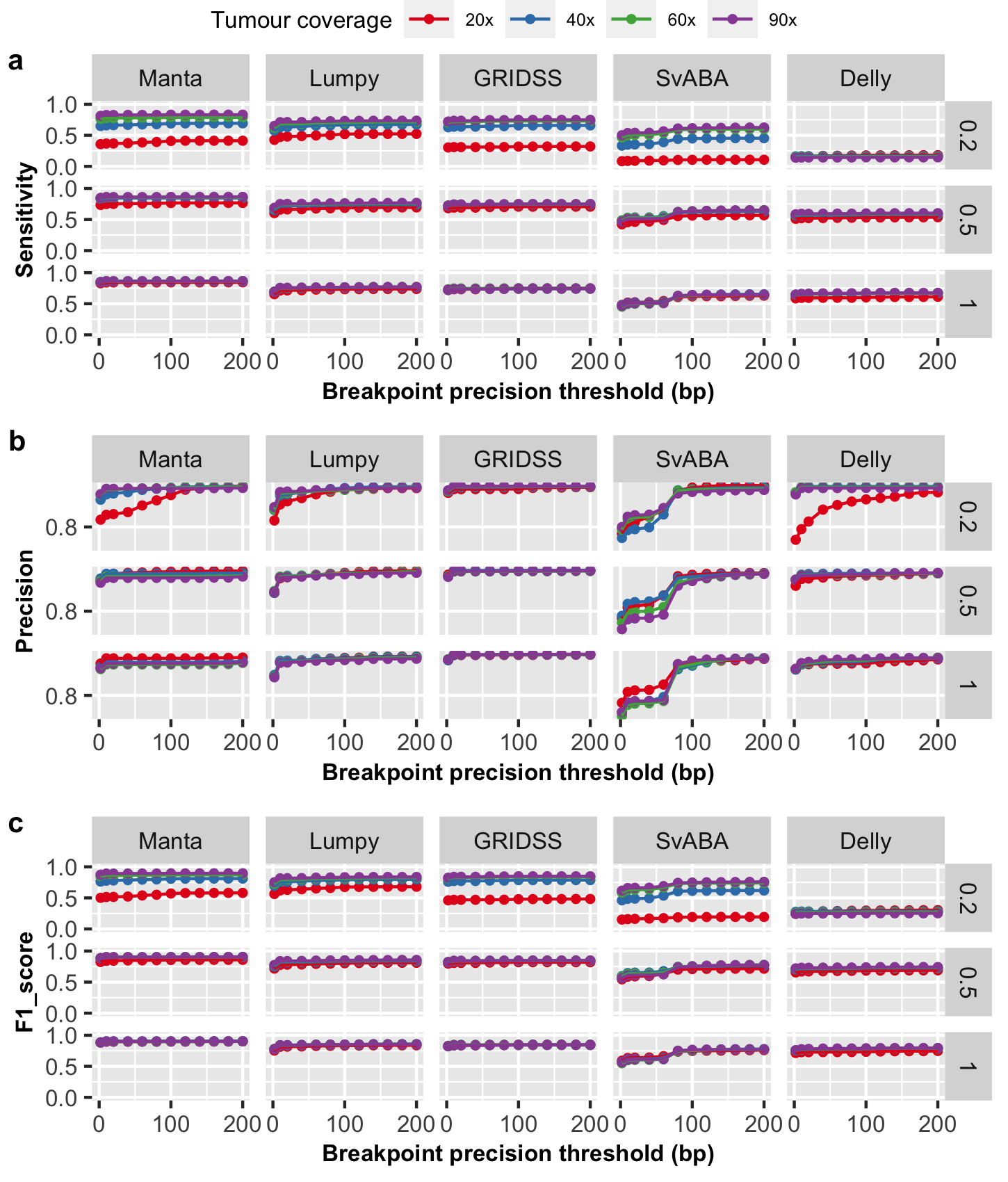

Supplement: S3 Fig — Shown are the effect of the interaction of breakpoint precision threshold and tumour coverage (by colour) on sensitivity (a) and precision in log scale (b) for five SV callers (Manta, Lumpy, GRIDSS, SvABA, Delly). Results are based on simulation data set with VAF of 0.2, 0.5 and 1, tumour coverage of 20x, 40x, 60x and 90x and normal coverage of 60x. (TIF) [file pone.0238108.s003.tif]

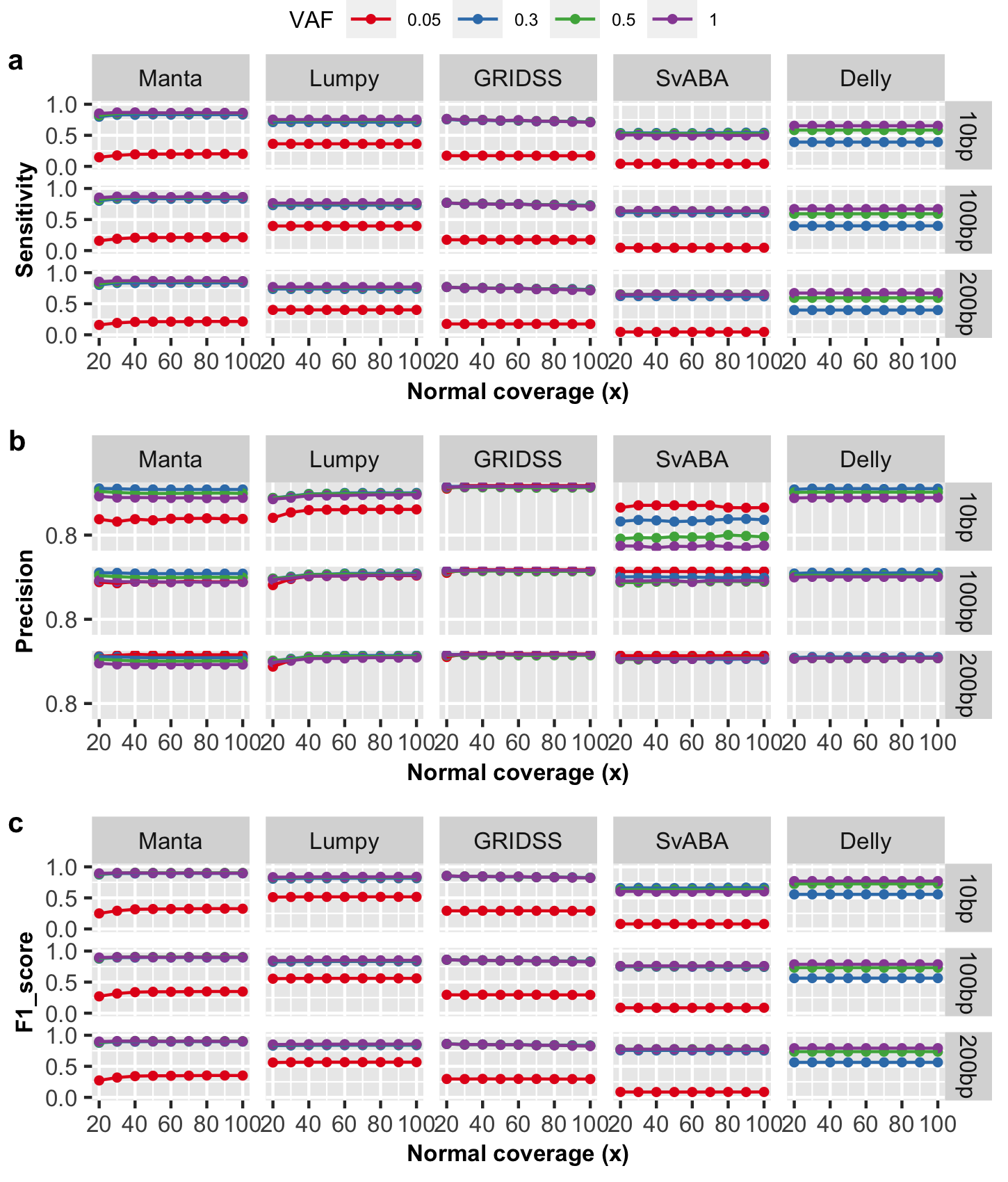

Supplement: S4 Fig — Shown are the effect of the interaction of normal coverage and VAF (by colour) on sensitivity (a) and precision in log scale (b) for five SV callers (Manta, Lumpy, GRIDSS, SvABA, Delly). Results are based on simulation data set with breakpoint precision threshold of 10bp, 100bp and 200bp, VAF of 0.05, 0.3 0.5 and 1 and tumour coverage of 60x. (TIF) [file pone.0238108.s004.tif]

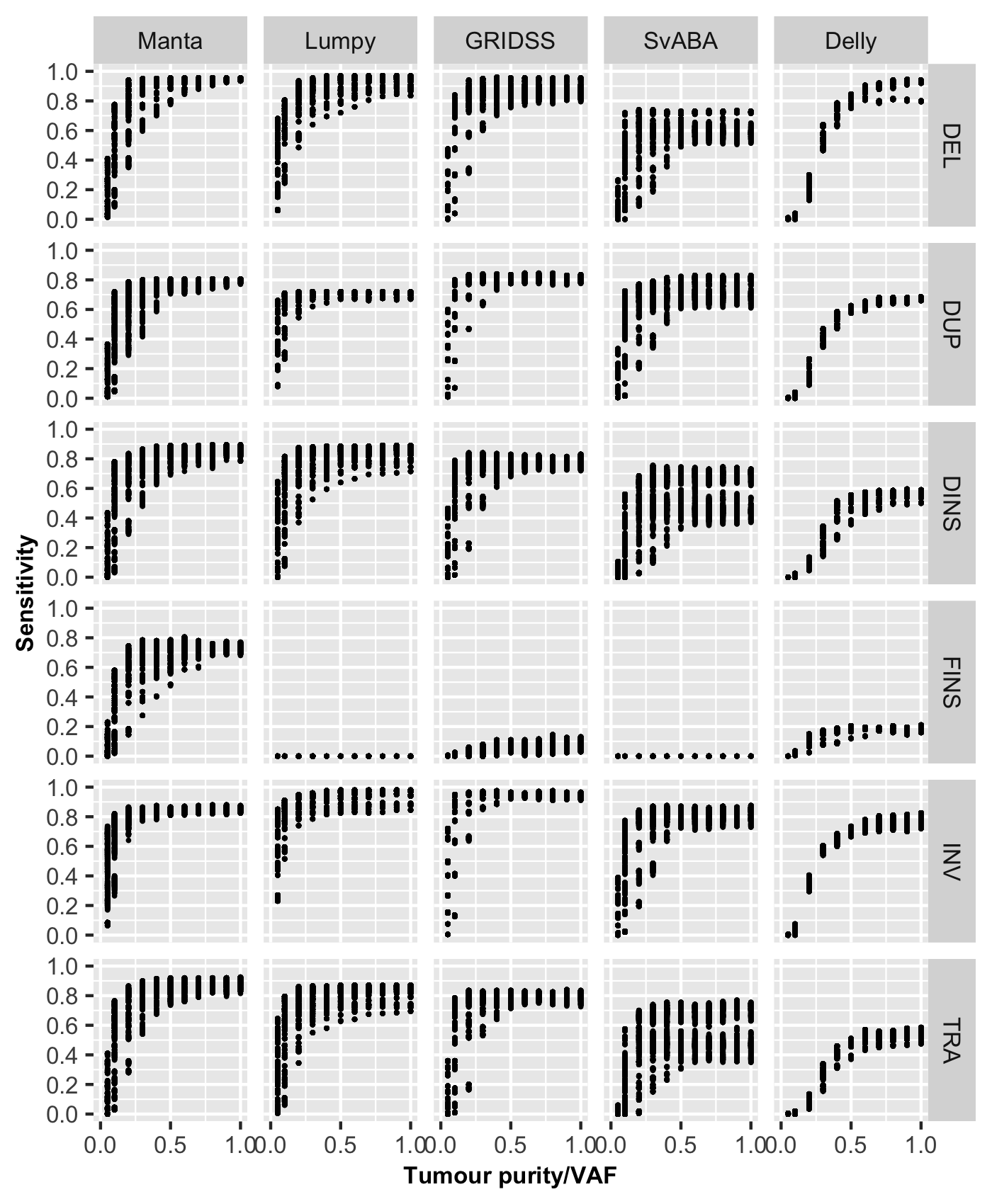

Supplement: S5 Fig — Shown are the effects of tumour purity/VAF on somatic SV type detection sensitivity for five SV callers (Manta, Lumpy, GRIDSS, SvABA, Delly). (TIF) [file pone.0238108.s005.tif]

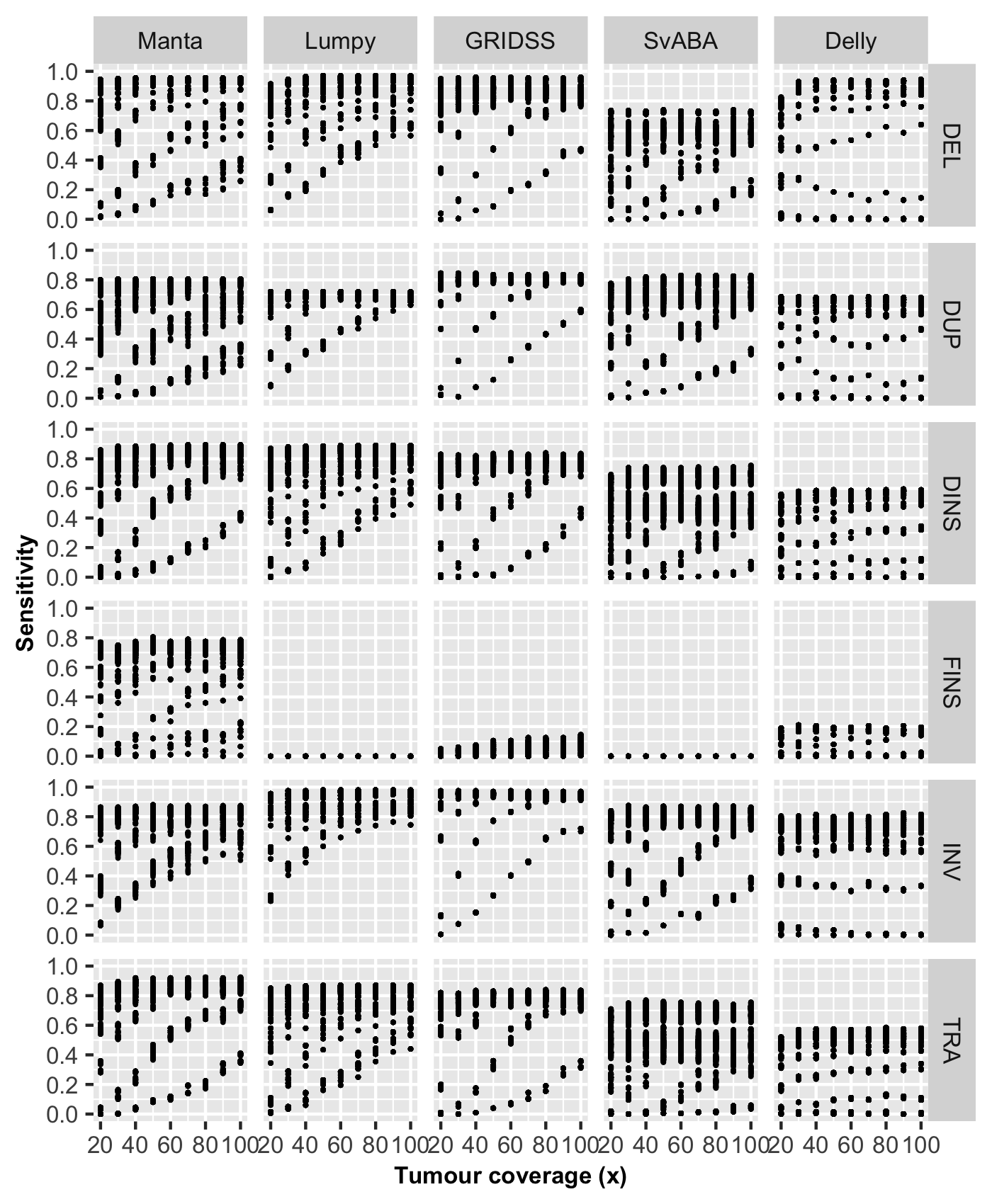

Supplement: S6 Fig — Shown are the effects of tumour coverage on somatic SV type detection sensitivity for five SV callers (Manta, Lumpy, GRIDSS, SvABA, Delly). (TIF) [file pone.0238108.s006.tif]

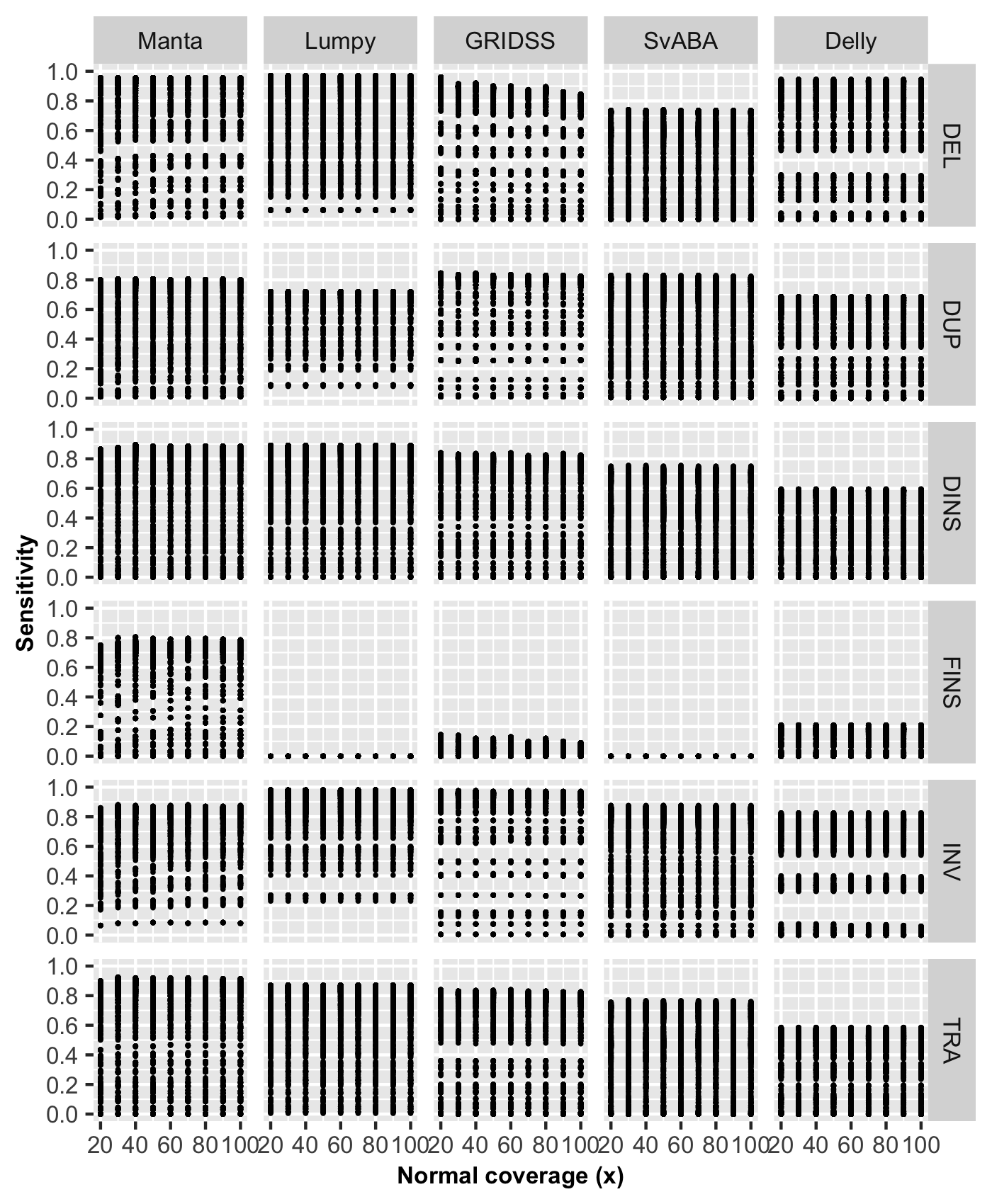

Supplement: S7 Fig — Shown are the effects of normal coverage on somatic SV type detection sensitivity for five SV callers (Manta, Lumpy, GRIDSS, SvABA, Delly). (TIF) [file pone.0238108.s007.tif]

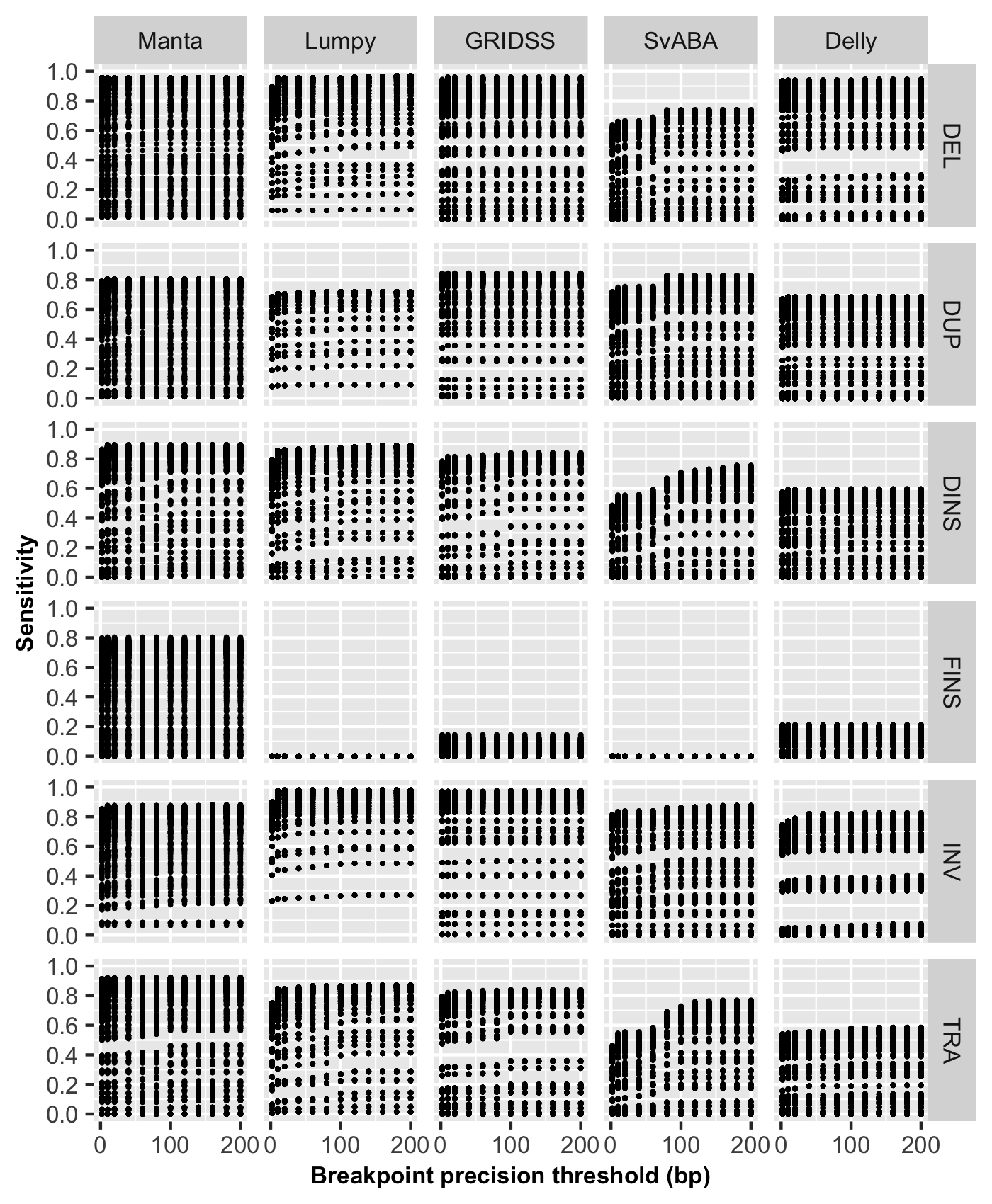

Supplement: S8 Fig — Shown are the effects of breakpoint precision threshold on somatic SV type detection sensitivity for five SV callers (Manta, Lumpy, GRIDSS, SvABA, Delly). (TIF) [file pone.0238108.s008.tif]

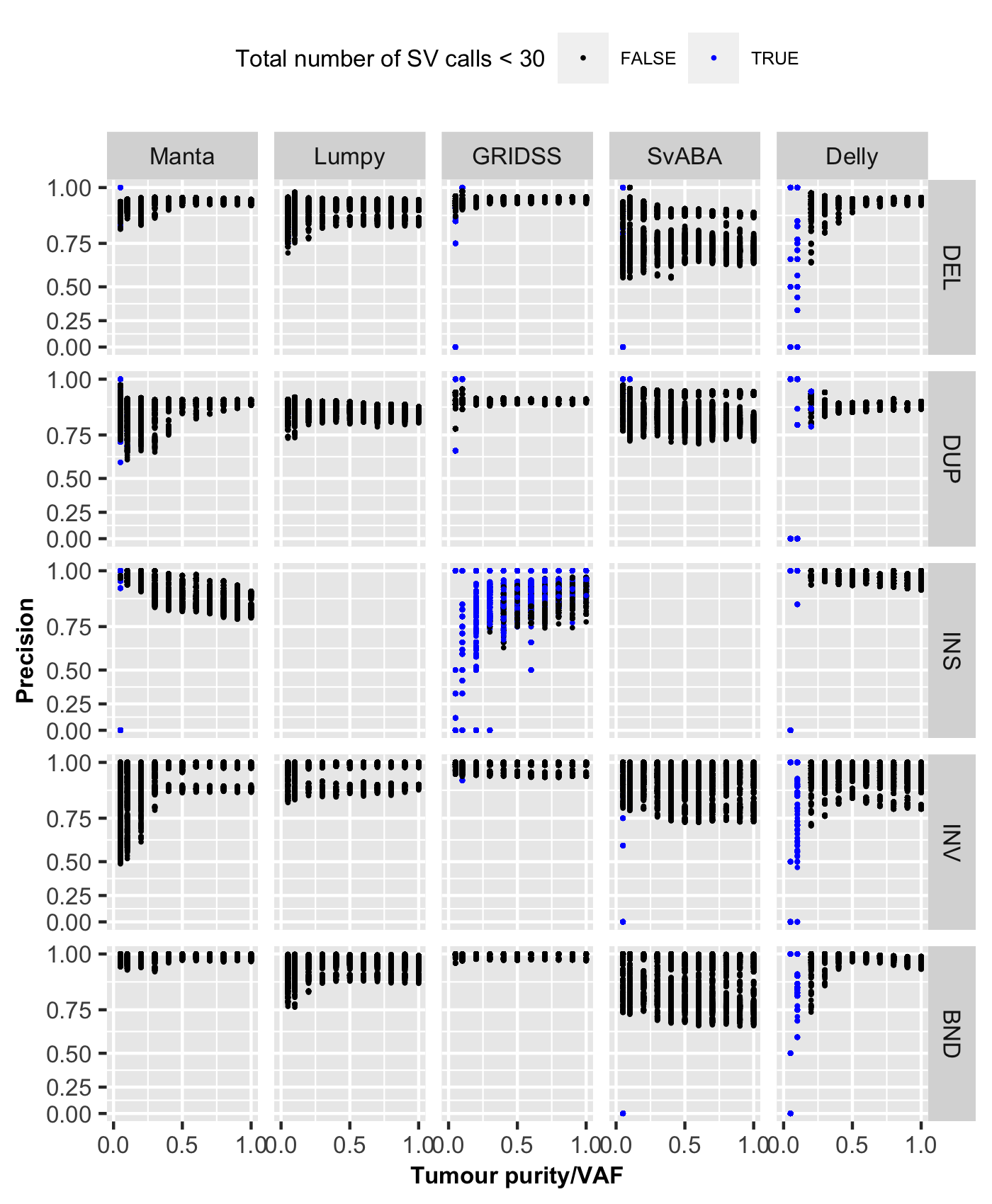

Supplement: S9 Fig — Shown are the effects of tumour purity/VAF on somatic SV type detection precision for five SV callers (Manta, Lumpy, GRIDSS, SvABA, Delly). INS is not detectable by Lumpy. Precision of DUP for SvABA shown can also be INS. (TIF) [file pone.0238108.s009.tif]

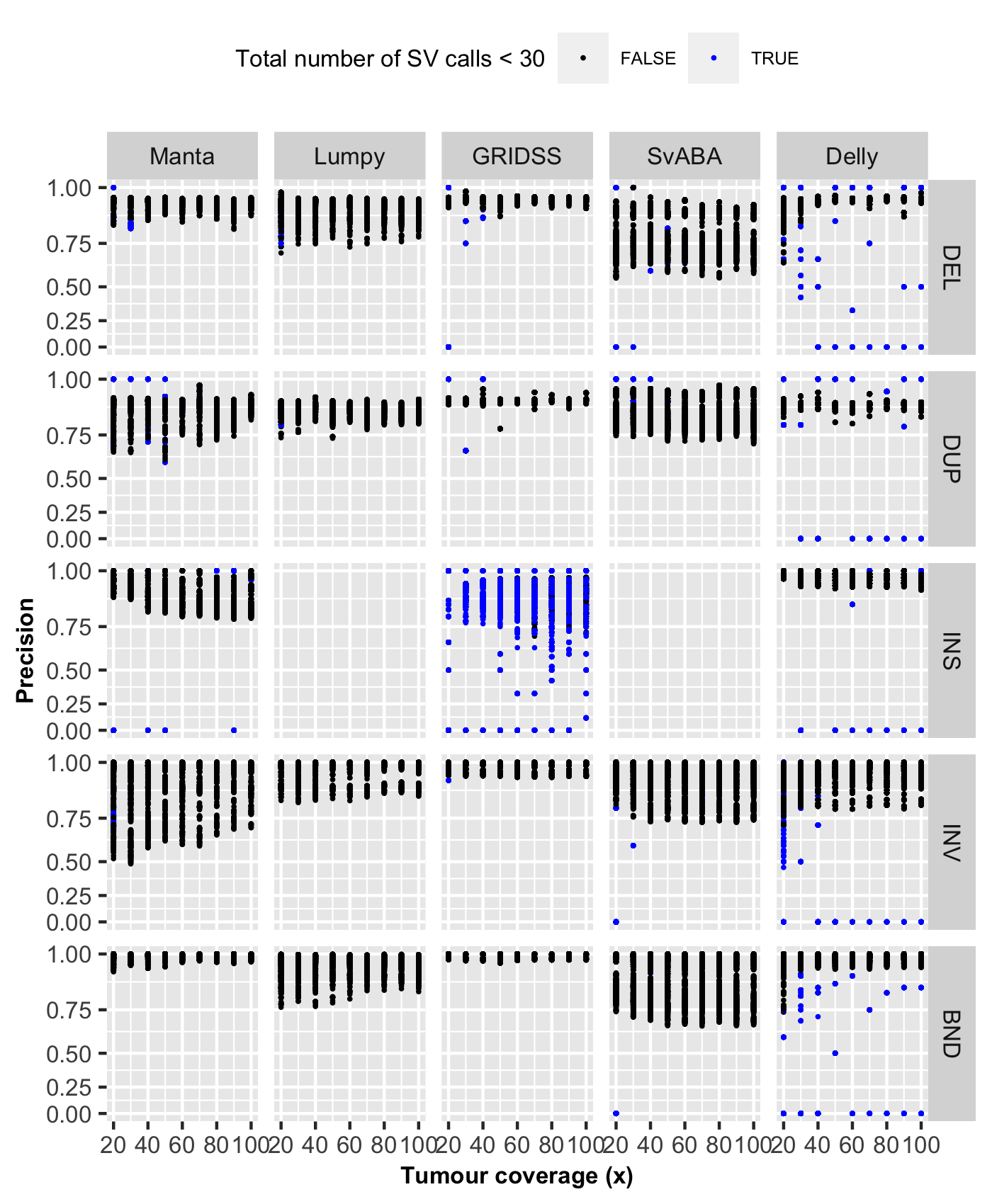

Supplement: S10 Fig — Shown are the effects of tumour coverage on somatic SV type detection precision for five SV callers (Manta, Lumpy, GRIDSS, SvABA, Delly). INS is not detectable by Lumpy. Precision of DUP for SvABA shown can also be INS. (TIF) [file pone.0238108.s010.tif]

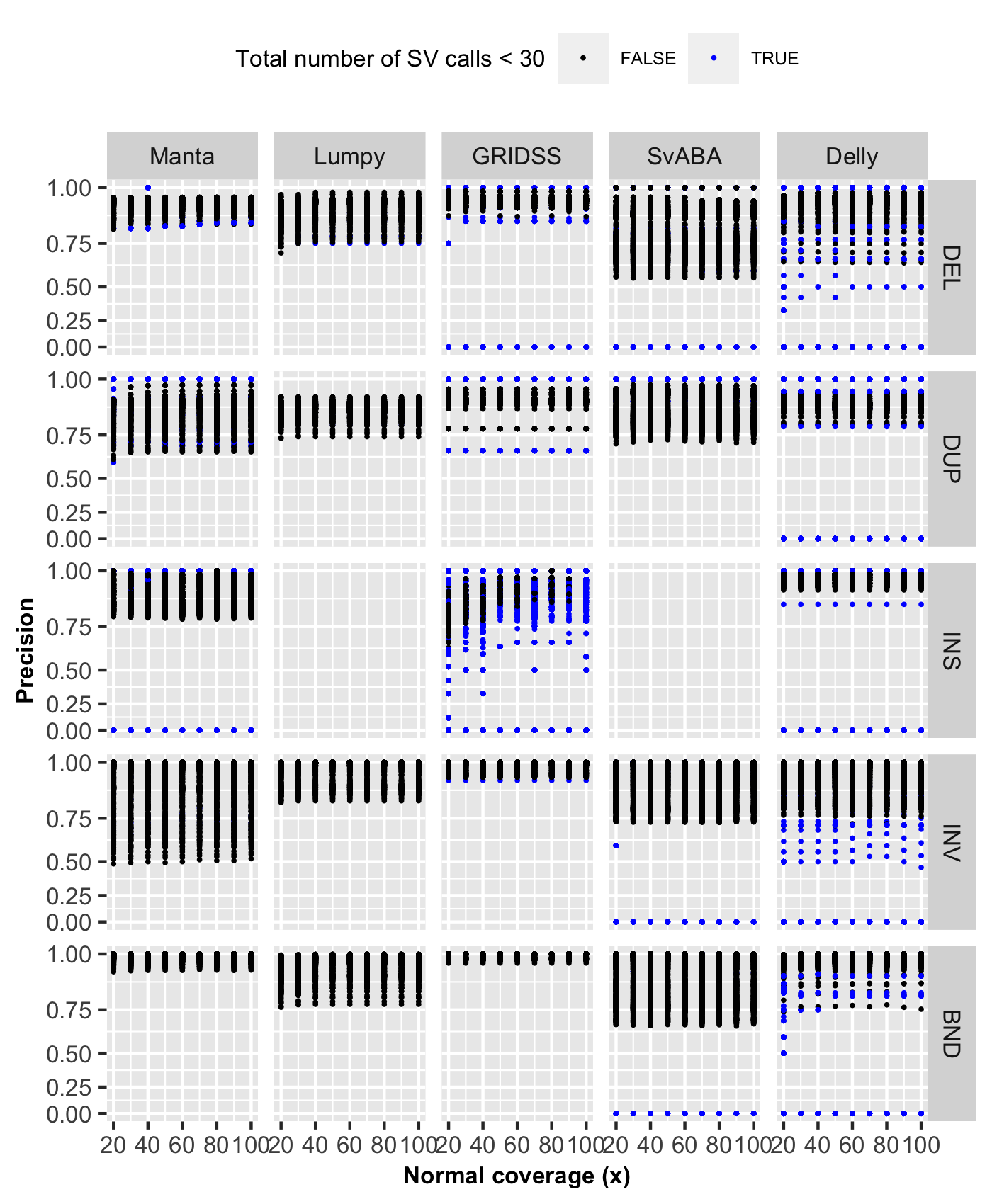

Supplement: S11 Fig — Shown are the effects of normal coverage on somatic SV type detection precision for five SV callers (Manta, Lumpy, GRIDSS, SvABA, Delly). INS is not detectable by Lumpy. Precision of DUP for SvABA shown can also be INS. (TIF) [file pone.0238108.s011.tif]

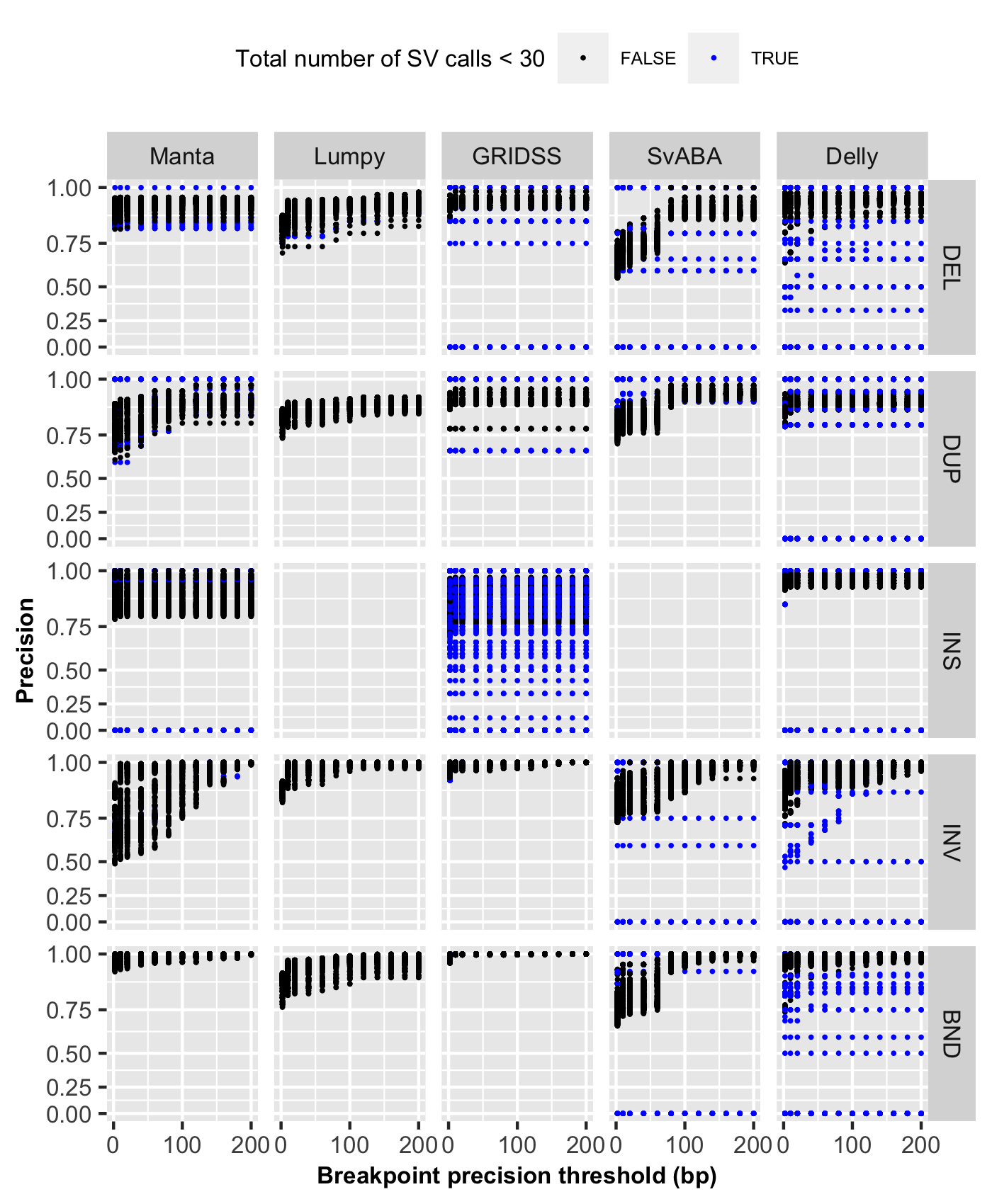

Supplement: S12 Fig — Shown are the effects of breakpoint precision threshold on somatic SV type detection precision for five SV callers (Manta, Lumpy, GRIDSS, SvABA, Delly). INS is not detectable by Lumpy. Precision of DUP for SvABA shown can also be INS. (TIF) [file pone.0238108.s012.tif]

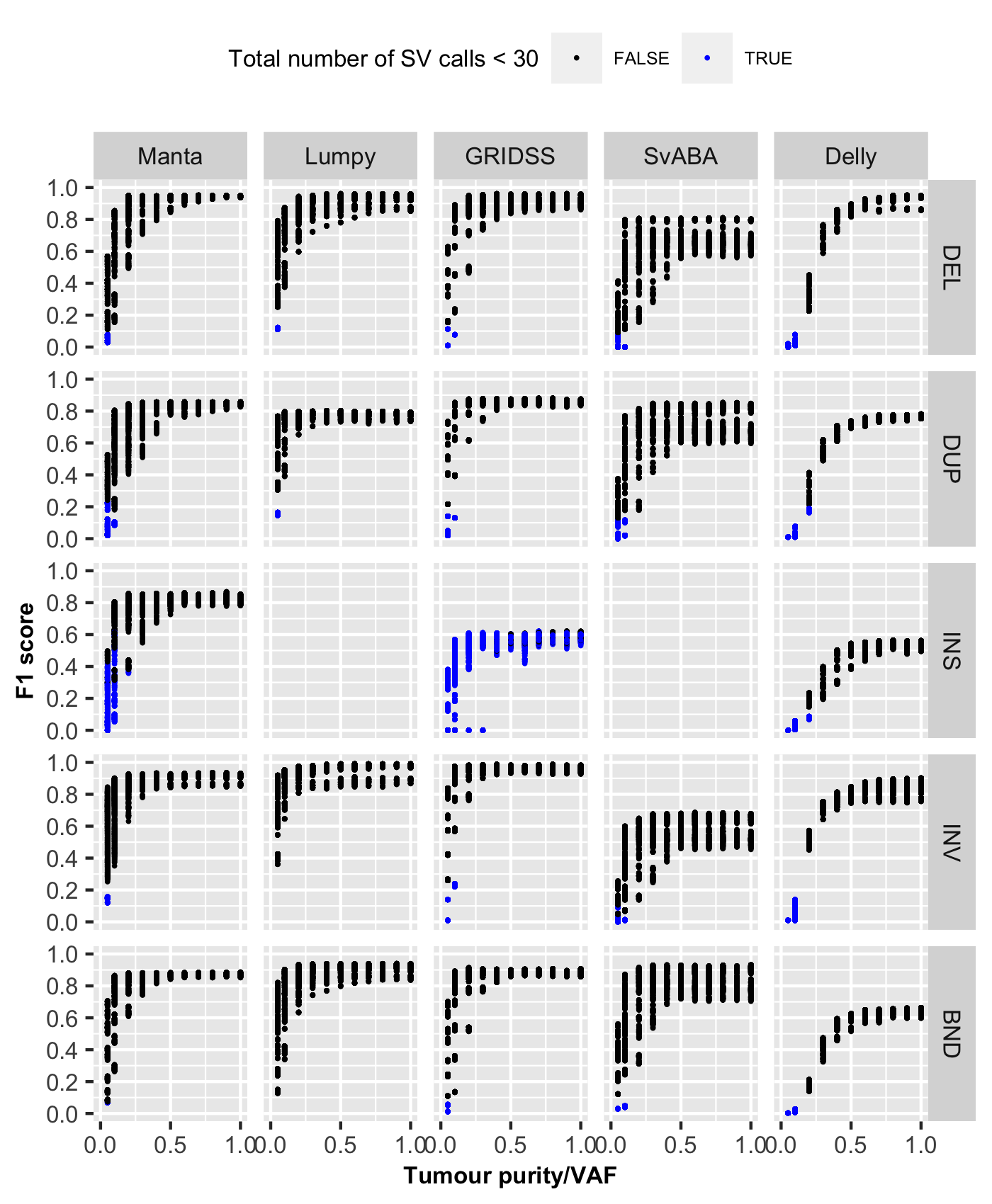

Supplement: S13 Fig — Shown are the effects of tumour purity/VAF on somatic SV type detection F1 score for five SV callers (Manta, Lumpy, GRIDSS, SvABA, Delly). INS is not detectable by Lumpy. F1 score of DUP for SvABA shown can also be INS. (TIF) [file pone.0238108.s013.tif]

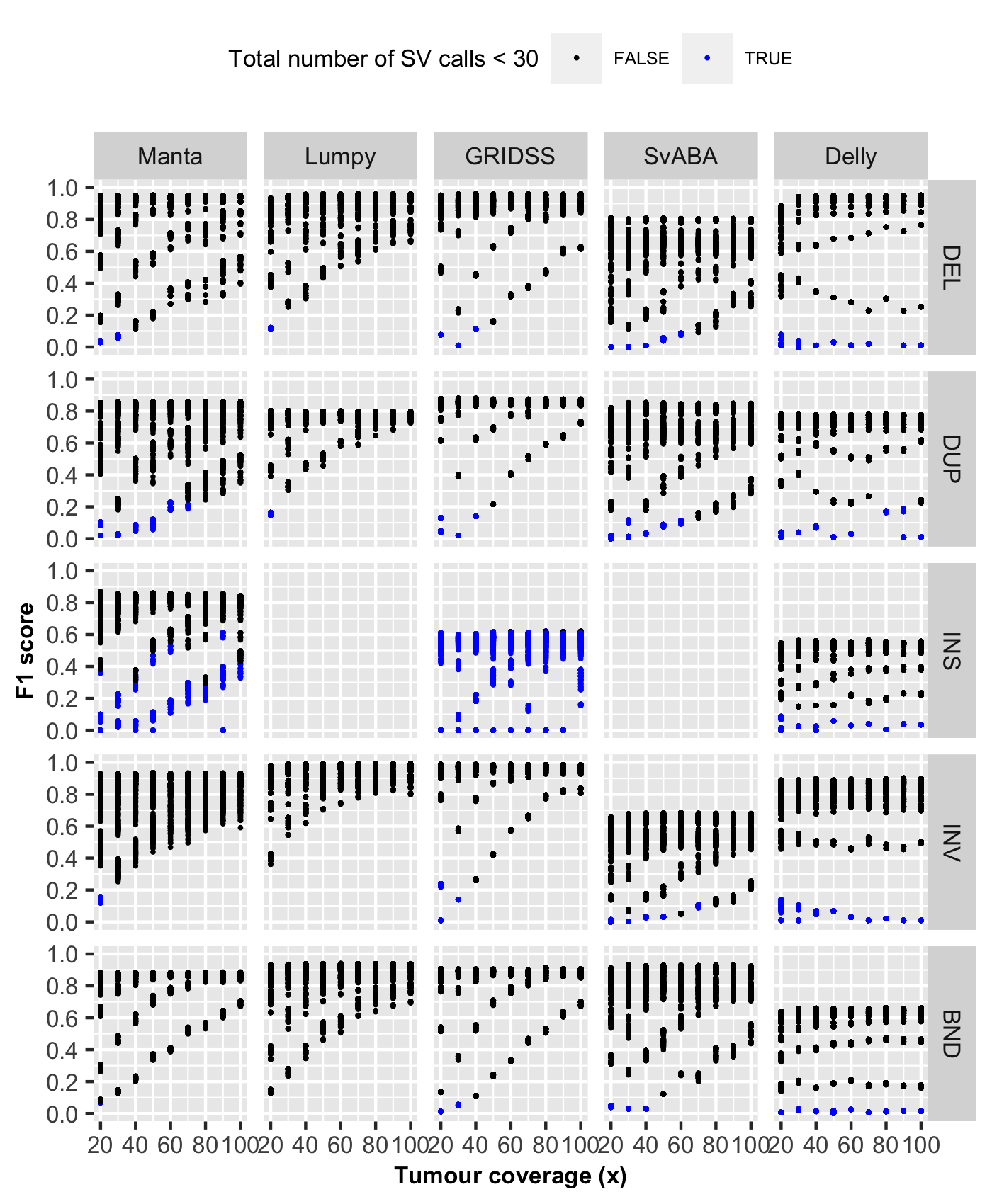

Supplement: S14 Fig — Shown are the effects of tumour coverage on somatic SV type detection F1 score for five SV callers (Manta, Lumpy, GRIDSS, SvABA, Delly). INS is not detectable by Lumpy. F1 score of DUP for SvABA shown can also be INS. (TIF) [file pone.0238108.s014.tif]

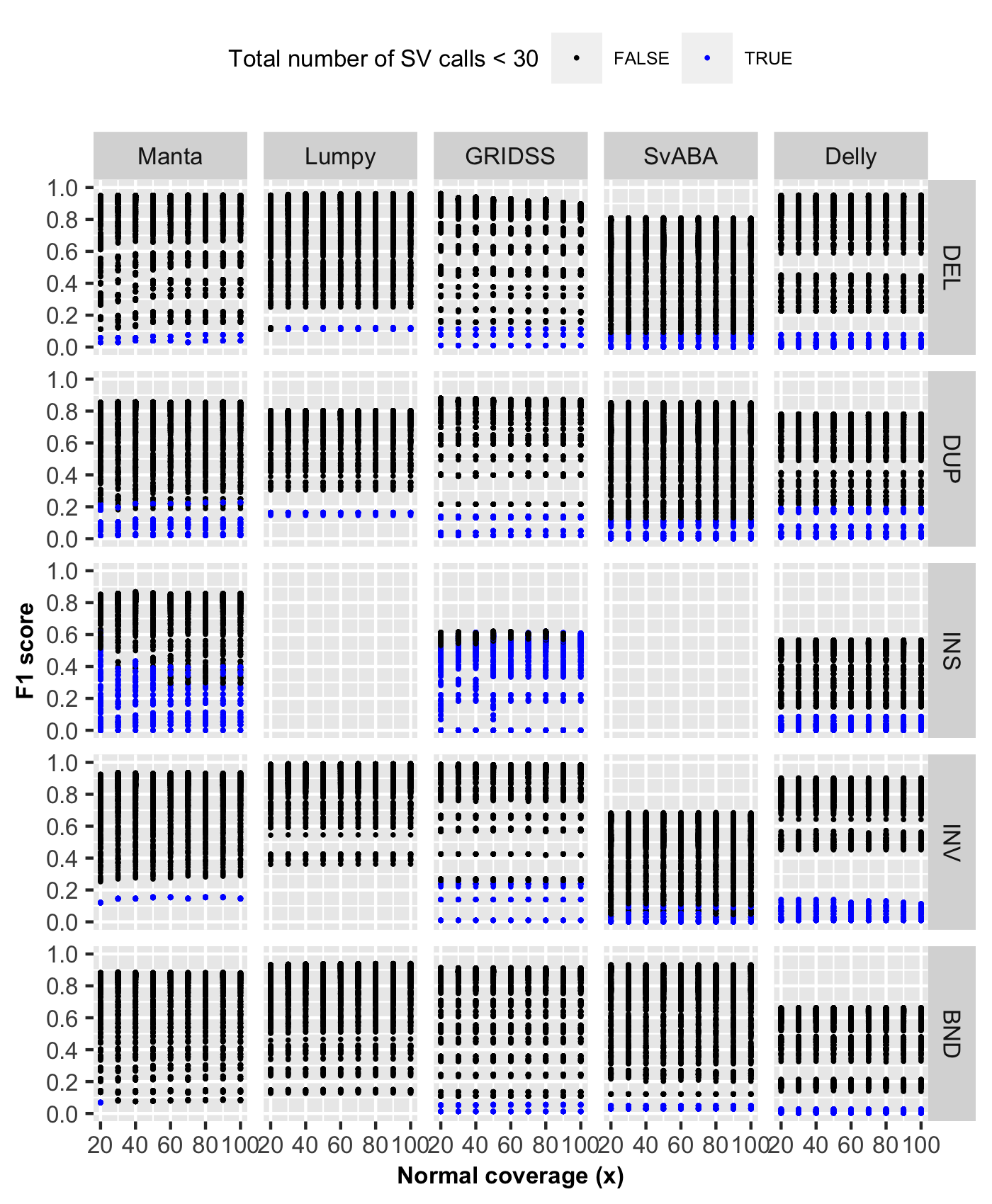

Supplement: S15 Fig — Shown are the effects of normal coverage on somatic SV type detection F1 score for five SV callers (Manta, Lumpy, GRIDSS, SvABA, Delly). INS is not detectable by Lumpy. F1 score of DUP for SvABA shown can also be INS. (TIF) [file pone.0238108.s015.tif]

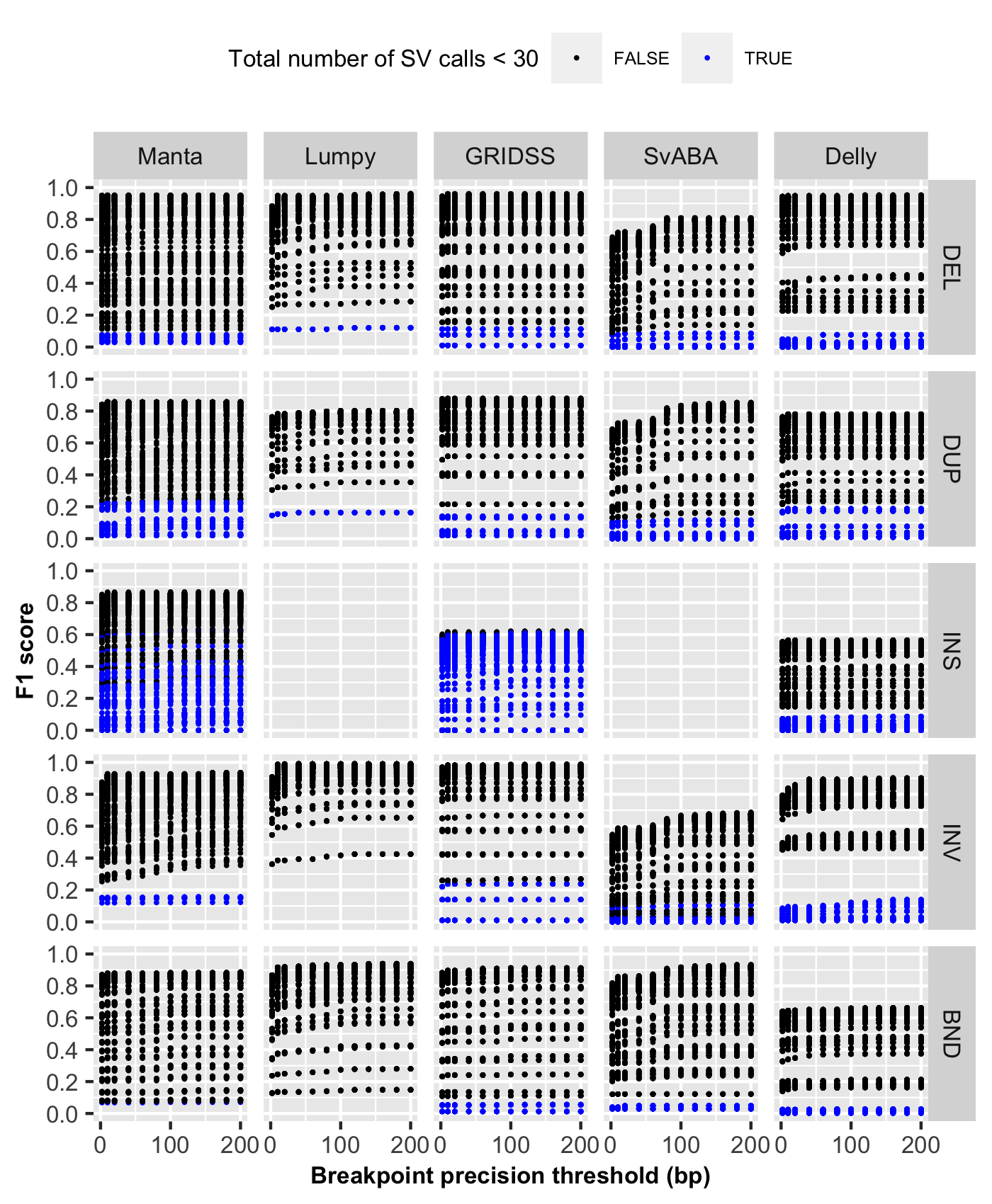

Supplement: S16 Fig — Shown are the effects of breakpoint precision threshold on somatic SV type detection F1 score for five SV callers (Manta, Lumpy, GRIDSS, SvABA, Delly). INS is not detectable by Lumpy. F1 score of DUP for SvABA shown can also be INS. (TIF) [file pone.0238108.s016.tif]
