# Supplementary material for: Shiny-SoSV: A web-based performance calculator for somatic structural variant detection
Source: PLoS One. 2020 Aug 27;15(8):e0238108. doi: 10.1371/journal.pone.0238108 (PMC7451576; doi:10.1371/journal.pone.0238108)
Supplement: S2 Table — (DOCX) [file pone.0238108.s018.docx]

**Table S2. Predictive model comparison and selection based on MAE**

| Error Estimate (MAE) | Models | Manta | Lumpy | GRIDSS | SvABA | Delly |
| --- | --- | --- | --- | --- | --- | --- |
| Sensitivity | (1) | 0.031 | 0.027 | 0.040 | 0.041 | 0.017 |
|  | (2) | **0.031** | 0.027 | 0.040 | 0.041 | 0.017 |
|  | (3) | 0.032 | 0.027 | 0.040 | **0.041** | 0.017 |
|  | (4) | 0.032 | 0.027 | 0.040 | 0.041 | 0.017 |
|  | (5) | 0.032 | **0.027** | 0.040 | 0.041 | 0.017 |
|  | (6) | 0.032 | 0.028 | **0.040** | 0.046 | **0.017** |
|  | (7) | 0.054 | 0.047 | 0.073 | 0.072 | 0.021 |
|  | (8) | 0.112 | 0.074 | 0.123 | 0.129 | 0.144 |
| Precision | (1) | 0.008 | 0.009 | 0.004 | 0.021 | 0.032 |
|  | (2) | 0.008 | **0.009** | 0.004 | 0.021 | **0.032** |
|  | (3) | **0.008** | 0.010 | 0.004 | 0.021 | 0.132 |
|  | (4) | 0.008 | 0.010 | 0.004 | 0.021 | 0.132 |
|  | (5) | 0.012 | 0.010 | **0.004** | **0.021** | 0.132 |
|  | (6) | 0.014 | 0.012 | 0.005 | 0.024 | 0.133 |
|  | (7) | 0.011 | 0.011 | 0.009 | 0.033 | 0.132 |
|  | (8) | 0.016 | 0.015 | 0.010 | 0.041 | 0.130 |
| F1 score | (1) | 0.029 | 0.025 | 0.037 | 0.045 | 0.015 |
|  | (2) | **0.029** | 0.025 | 0.037 | 0.046 | 0.015 |
|  | (3) | 0.030 | 0.025 | 0.037 | **0.045** | 0.015 |
|  | (4) | 0.030 | 0.025 | 0.037 | 0.046 | 0.015 |
|  | (5) | 0.030 | **0.024** | 0.037 | 0.046 | **0.015** |
|  | (6) | 0.030 | 0.026 | **0.037** | 0.052 | 0.015 |
|  | (7) | 0.052 | 0.044 | 0.071 | 0.080 | 0.016 |
|  | (8) | 0.104 | 0.065 | 0.116 | 0.141 | 0.130 |
