# Supplementary material for: Shiny-SoSV: A web-based performance calculator for somatic structural variant detection
Source: PLoS One. 2020 Aug 27;15(8):e0238108. doi: 10.1371/journal.pone.0238108 (PMC7451576; doi:10.1371/journal.pone.0238108)
Supplement: S3 Table — (DOCX) [file pone.0238108.s019.docx]

**Table S3 The parametric coefficients and approximate significance of smooth terms of the selected GAM models**

| **Sensitivity Models** | | | | |
| --- | --- | --- | --- | --- |
| Manta | | | | |
| Nominal variables | Parametric coefficients | | | |
|  | Estimate | Standard errors | z-value | Pr(>\|z\|)^1^ |
| Intercept | 1.1521 | 0.0021 | 555 | <2e-16 *** |
| Smooth terms | Approximate significance of smooth terms | | | |
|  | edf^2^ | Ref.df^3^ | Chi Square | p-value^1^ |
| te(VAF, Tumour coverage) | 23.7 | 24 | 66235 | <2e-16 *** |
| te(VAF, *T*) | 12.8 | 20 | 404 | <2e-16 *** |
| te(VAF, Normal coverage) | 19.1 | 20 | 1667 | <2e-16 *** |
| Lumpy | | | | |
| Nominal variables | Parametric coefficients | | | |
|  | Estimate | Standard errors | z-value | Pr(>\|z\|)^1^ |
| Intercept | 0.7699 | 0.0038 | 228 | <2e-16 *** |
| Normal coverage | 0.0000 | 0.0001 | 0.034 | 0.973 |
| Smooth terms | Approximate significance of smooth terms | | | |
|  | edf^2^ | Ref.df^3^ | Chi Square | p-value^1^ |
| te(VAF, Tumour coverage) | 23.8 | 24 | 174656 | <2e-16 *** |
| te(VAF, Normal coverage) | 19.7 | 20 | 5162 | <2e-16 *** |
| GRIDSS | | | | |
| Nominal variables | Parametric coefficients | | | |
|  | Estimate | Standard errors | z-value | Pr(>\|z\|)^1^ |
| Intercept | 0.6788 | 0.0063 | 107.4 | <2e-16 *** |
| *T* | 0.0004 | 0.0000 | 11.4 | <2e-16 *** |
| Normal coverage | -0.0021 | 0.0001 | -24.8 | <2e-16 *** |
| Smooth terms | Approximate significance of smooth terms | | | |
|  | edf^2^ | Ref.df^3^ | Chi Square | p-value^1^ |
| te(VAF, Tumour coverage) | 23.8 | 24 | 111306 | <2e-16 *** |
| SvABA | | | | |
| Nominal variables | Parametric coefficients | | | |
|  | Estimate | Standard errors | z-value | Pr(>\|z\|)^1^ |
| Intercept | -0.1966 | 0.0057 | -35.34 | <2e-16 *** |
| Normal coverage | -0.0000 | 0.0001 | -0.41 | 0.685 |
| Smooth terms | Approximate significance of smooth terms | | | |
|  | Edf^2^ | Ref.df^3^ | Chi Square | p-value^1^ |
| te(VAF, Tumour coverage) | 23.8 | 24 | 28278 | <2e-16 *** |
| te(VAF, *T*) | 18.9 | 20 | 2973 | <2e-16 *** |
| te(Tumour coverage, *T*) | 11.9 | 16 | 189 | <2e-16 *** |
| Delly | | | | |
| Nominal variables | Parametric coefficients | | | |
|  | Estimate | Standard errors | z-value | Pr(>\|z\|)^1^ |
| Intercept | -0.9408 | 0.0051 | -184.27 | <2e-16 *** |
| *T* | 0.0004 | 0.0000 | 17.29 | <2e-16 *** |
| Normal coverage | -0.0000 | 0.0001 | -0.08 | 0.93 |
| Smooth terms | Approximate significance of smooth terms | | | |
|  | edf^2^ | Ref.df^3^ | Chi Square | p-value^1^ |
| te(VAF, Tumour coverage) | 23.9 | 24 | 283808 | <2e-16 *** |
| **Precision Models** | | | | |
| Manta | | | | |
| Nominal variables | Parametric coefficients | | | |
|  | Estimate | Standard errors | z-value | Pr(>\|z\|)^1^ |
| Intercept | 3.051 | 0.0045 | 673.32 | <2e-16 *** |
| Normal coverage | -0.0004 | 0.0001 | -6.404 | 7.62-12 *** |
| Smooth terms | Approximate significance of smooth terms | | | |
|  | edf^2^ | Ref.df^3^ | Chi Square | p-value^1^ |
| te(VAF, Tumour coverage) | 23.88 | 24 | 19440 | <2e-16 *** |
| te(VAF, *T*) | 19.18 | 20 | 16974 | <2e-16 *** |
| te(Tumour coverage, *T*) | 15.49 | 16 | 2541 | <2e-16 *** |
| Lumpy | | | | |
| Nominal variables | Parametric coefficients | | | |
|  | Estimate | Standard errors | z-value | Pr(>\|z\|)^1^ |
| Intercept | 3.01 | 0.0020 | 1517 | <2e-16 *** |
| Smooth terms | Approximate significance of smooth terms | | | |
|  | edf^2^ | Ref.df^3^ | Chi Square | p-value^1^ |
| te(VAF, Tumour coverage) | 22.90 | 23.84 | 9111 | <2e-16 *** |
| te(VAF, *T*) | 19.96 | 20 | 51358 | <2e-16 *** |
| Te(VAF, Normal coverage) | 18.67 | 20 | 3913 | <2e-16 *** |
| GRIDSS | | | | |
| Nominal variables | Parametric coefficients | | | |
|  | Estimate | Standard errors | z-value | Pr(>\|z\|)^1^ |
| Intercept | 3.649 | 0.0060 | 610.768 | <2e-16 *** |
| Normal coverage | 0.0007 | 0.0001 | 7.444 | 9.74e-14 *** |
| Smooth terms | Approximate significance of smooth terms | | | |
|  | edf^2^ | Ref.df^3^ | Chi Square | p-value^1^ |
| te(VAF, Tumour coverage) | 23.96 | 24 | 6327 | <2e-16 *** |
| te(Tumour coverage, *T*) | 19.78 | 20 | 4226 | <2e-16 *** |
| SvABA | | | | |
| Nominal variables | Parametric coefficients | | | |
|  | Estimate | Standard errors | z-value | Pr(>\|z\|)^1^ |
| Intercept | 2.4646 | 0.0071 | 347.885 | <2e-16 *** |
| Normal coverage | 0.0003 | 0.0001 | 3.114 | 0.00185 ** |
| Smooth terms | Approximate significance of smooth terms | | | |
|  | edf^2^ | Ref.df^3^ | Chi Square | p-value^1^ |
| te(VAF, Tumour coverage) | 23.98 | 24 | 23078 | <2e-16 *** |
| te(Tumour coverage, *T*) | 19.92 | 20 | 82566 | <2e-16 *** |
| Delly | | | | |
| Nominal variables | Parametric coefficients | | | |
|  | Estimate | Standard errors | z-value | Pr(>\|z\|)^1^ |
| Intercept | 3.2090 | 0.007515 | 427 | <2e-16 *** |
| Smooth terms | Approximate significance of smooth terms | | | |
|  | edf^2^ | Ref.df^3^ | Chi Square | p-value^1^ |
| te(VAF, Tumour coverage) | 23.67 | 23.98 | 6861.9 | <2e-16 *** |
| te(VAF, *T*) | 516.57 | 20 | 492.5 | <2e-16 *** |
| te(VAF, Normal coverage) | 19.98 | 20 | 22723.1 | <2e-16 *** |
| **F1 score Models** | | | | |
| Manta | | | | |
| Nominal variables | Parametric coefficients | | | |
|  | Estimate | Standard errors | z-value | Pr(>\|z\|)^1^ |
| Intercept | 1.6440 | 0.0023 | 713.4 | <2e-16 *** |
| Smooth terms | Approximate significance of smooth terms | | | |
|  | edf^2^ | Ref.df^3^ | Chi Square | p-value^1^ |
| te(VAF, Tumour coverage) | 23.69 | 24 | 65128.6 | <2e-16 *** |
| te(VAF, *T*) | 16.78 | 20 | 797.7 | <2e-16 *** |
| te(VAF, Normal coverage) | 18.34 | 16 | 1200.7 | <2e-16 *** |
| Lumpy | | | | |
| Nominal variables | Parametric coefficients | | | |
|  | Estimate | Standard errors | z-value | Pr(>\|z\|)^1^ |
| Intercept | 1.322 | 0.0038 | 351.457 | <2e-16 *** |
| Normal coverage | 0.0004 | 0.0001 | 6.966 | 3.27e-12*** |
| Smooth terms | Approximate significance of smooth terms | | | |
|  | edf^2^ | Ref.df^3^ | Chi Square | p-value^1^ |
| te(VAF, Tumour coverage) | 23.87 | 24 | 177021 | <2e-16 *** |
| te(Tumour coverage, *T*) | 19.81 | 20 | 7408 | <2e-16 *** |
| GRIDSS | | | | |
| Nominal variables | Parametric coefficients | | | |
|  | Estimate | Standard errors | z-value | Pr(>\|z\|)^1^ |
| Intercept | 1.274 | 0.0071 | 180.20 | <2e-16 *** |
| *T* | 0.0004 | 0.00004 | 11.81 | <2e-16 *** |
| Normal coverage | -0.0017 | 0.0001 | -18.44 | <2e-16 *** |
| Smooth terms | Approximate significance of smooth terms | | | |
|  | edf^2^ | Ref.df^3^ | Chi Square | p-value^1^ |
| te(VAF, Tumour coverage) | 23.87 | 24 | 177021 | <2e-16 *** |
| SvABA | | | | |
| Nominal variables | Parametric coefficients | | | |
|  | Estimate | Standard errors | z-value | Pr(>\|z\|)^1^ |
| Intercept | 0.3556 | 0.0062 | 57.699 | <2e-16 *** |
| Normal coverage | -0.00001 | 0.0001 | -0.103 | 0.918 |
| Smooth terms | Approximate significance of smooth terms | | | |
|  | edf^2^ | Ref.df^3^ | Chi Square | p-value^1^ |
| te(VAF, Tumour coverage) | 23.71 | 23.95 | 28870 | <2e-16 *** |
| te(VAF, *T*) | 19.16 | 20 | 4002.5 | <2e-16 *** |
| te(Tumour coverage, *T*) | 12.93 | 16 | 317.9 | <2e-16 *** |
| Delly | | | | |
| Nominal variables | Parametric coefficients | | | |
|  | Estimate | Standard errors | z-value | Pr(>\|z\|)^1^ |
| Intercept | 0.1222 | 0.0030 | 40.888 | <2e-16 *** |
| Normal coverage | 0.00002 | 0.00004 | 0.383 | 0.702 |
| Smooth terms | Approximate significance of smooth terms | | | |
|  | edf^2^ | Ref.df^3^ | Chi Square | p-value^1^ |
| te(VAF, Tumour coverage) | 23.79 | 23.98 | 6888806 | <2e-16 *** |
| te(Tumour coverage, *T*) | 17.48 | 20 | 1137 | <2e-16 *** |

^1^Significanct code: 0: ***; 0.001: **.

^2^edf: estimated degree of freedom.

^3^Ref.df: estimated degree of freedom for reference.
