# Supplementary material for: Shiny-SoSV: A web-based performance calculator for somatic structural variant detection
Source: PLoS One. 2020 Aug 27;15(8):e0238108. doi: 10.1371/journal.pone.0238108 (PMC7451576; doi:10.1371/journal.pone.0238108)
Supplement: S4 Table — (DOCX) [file pone.0238108.s020.docx]

**Table S4. Predictive model comparison and selection for each SV type**

| Caller | | SV types | Models | | | | | | | | | | | | | | |
| --- | --- | --- | --- | --- | --- | --- | --- | --- | --- | --- | --- | --- | --- | --- | --- | --- | --- |
|  |  |  | (1) | | (2) | | (3) | | (4) | | (5) | | (6) | | (7) | | (8) |
| **Sensitivity** | | | | | | | | | | | | | | | | | |
| Manta | | DEL | 0.033 | | **0.033** | | 0.034 | | 0.034 | | 0.034 | | 0.034 | | 0.054 | | 0.139 |
|  |  | DUP | **0.052** | | 0.053 | | 0.054 | | 0.054 | | 0.054 | | 0.055 | | 0.083 | | 0.150 |
|  |  | DINS | **0.063** | | 0.064 | | 0.064 | | 0.064 | | 0.064 | | 0.064 | | 0.100 | | 0.178 |
|  |  | FINS | 0.059 | | **0.059** | | 0.072 | | 0.072 | | 0.072 | | 0.072 | | 0.102 | | 0.183 |
|  |  | INV | 0.040 | | **0.040** | | 0.041 | | 0.041 | | 0.041 | | 0.041 | | 0.067 | | 0.098 |
|  |  | TRA | 0.058 | | **0.058** | | 0.059 | | 0.059 | | 0.059 | | 0.059 | | 0.096 | | 0.192 |
| Lumpy | | DEL | 0.032 | | 0.033 | | **0.032** | | 0.033 | | 0.033 | | 0.034 | | 0.044 | | 0.093 |
|  |  | DUP | 0.035 | | 0.035 | | 0.035 | | 0.035 | | 0.035 | | **0.035** | | 0.067 | | 0.086 |
|  |  | DINS | 0.054 | | 0.054 | | 0.054 | | **0.054** | | 0.055 | | 0.056 | | 0.095 | | 0.144 |
|  |  | INV | 0.030 | | 0.031 | | **0.030** | | 0.031 | | 0.034 | | 0.036 | | 0.042 | | 0.065 |
|  |  | TRA | 0.048 | | 0.048 | | 0.048 | | **0.048** | | 0.049 | | 0.052 | | 0.080 | | 0.131 |
| GRIDSS | | DEL | 0.052 | | **0.052** | | 0.055 | | 0.055 | | 0.055 | | 0.055 | | 0.107 | | 0.190 |
|  |  | DUP | 0.055 | | 0.055 | | 0.055 | | 0.055 | | 0.055 | | **0.055** | | 0.098 | | 0.149 |
|  |  | DINS | 0.068 | | 0.068 | | 0.068 | | **0.068** | | 0.069 | | 0.069 | | 0.131 | | 0.209 |
|  |  | FINS | 0.011 | | 0.011 | | 0.011 | | 0.011 | | 0.011 | | **0.011** | | 0.017 | | 0.032 |
|  |  | INV | 0.038 | | 0.038 | | 0.038 | | 0.038 | | 0.038 | | **0.038** | | 0.082 | | 0.158 |
|  |  | TRA | 0.069 | | 0.069 | | 0.069 | | **0.069** | | 0.070 | | 0.070 | | 0.131 | | 0.219 |
| SvABA | | DEL | 0.065 | | 0.065 | | 0.065 | | **0.065** | | 0.066 | | 0.071 | | 0.134 | | 0.211 |
|  |  | DUP | 0.060 | | 0.061 | | **0.060** | | 0.061 | | 0.062 | | 0.066 | | 0.096 | | 0.157 |
|  |  | DINS | 0.075 | | 0.076 | | **0.075** | | 0.076 | | 0.076 | | 0.084 | | 0.121 | | 0.221 |
|  |  | INV | 0.055 | | 0.055 | | 0.055 | | 0.055 | | **0.055** | | 0.056 | | 0.113 | | 0.186 |
|  |  | TRA | 0.069 | | 0.071 | | **0.069** | | 0.071 | | 0.072 | | 0.080 | | 0.122 | | 0.218 |
| Delly | | DEL | 0.027 | | 0.027 | | 0.027 | | 0.027 | | 0.027 | | **0.027** | | 0.036 | | 0.199 |
|  |  | DUP | 0.028 | | 0.028 | | 0.028 | | 0.028 | | 0.028 | | 0**.028** | | 0.032 | | 0.181 |
|  |  | DINS | 0.022 | | 0.023 | | 0.022 | | 0.023 | | **0.022** | | 0.023 | | 0.031 | | 0.163 |
|  |  | FINS | 0.027 | | 0.027 | | 0.027 | | 0.027 | | 0.027 | | **0.027** | | 0.030 | | 0.086 |
|  |  | INV | 0.049 | | 0.049 | | 0.049 | | **0.049** | | 0.050 | | 0.050 | | 0.051 | | 0.210 |
|  |  | TRA | 0.022 | | 0.022 | | 0.022 | | 0.022 | | 0.022 | | **0.022** | | 0.026 | | 0.157 |
| **Precision** | | | | | | | | | | | | | | | | | |
| Manta | DEL | | 0.003 | | 0.003 | | 0.003 | | 0.003 | | 0.003 | | **0.003** | | 0.004 | | 0.004 |
|  | DUP | | 0.005 | | 0.005 | | 0.005 | | 0.005 | | **0.005** | | 0.006 | | 0.006 | | 0.006 |
|  | INS | | 0.012 | | **0.012** | | 0.013 | | 0.013 | | 0.013 | | 0.013 | | 0.016 | | 0.018 |
|  | INV | | 0.014 | | 0.014 | | 0.014 | | 0.014 | | 0.014 | | 0.021 | | **0.014** | | 0.021 |
|  | BND | | 0.005 | | 0.005 | | 0.005 | | 0.005 | | 0.005 | | 0.005 | | **0.004** | | 0.005 |
| Lumpy | DEL | | 0.014 | | 0.014 | | 0.014 | | 0.014 | | 0.014 | | 0.017 | | **0.014** | | 0.017 |
|  | DUP | | 0.007 | | 0.007 | | 0.008 | | 0.008 | | 0.008 | | 0.011 | | **0.008** | | 0.011 |
|  | INV | | 0.014 | | 0.014 | | 0.013 | | 0.013 | | 0.013 | | 0.025 | | **0.013** | | 0.025 |
|  | BND | | 0.010 | | **0.010** | | 0.011 | | 0.011 | | 0.011 | | 0.013 | | 0.011 | | 0.013 |
| GRIDSS | DEL | | 0.003 | | 0.003 | | 0.003 | | 0.003 | | 0.003 | | 0.004 | | **0.003** | | 0.004 |
|  | DUP | | 0.002 | | 0.002 | | 0.002 | | 0.002 | | 0.002 | | **0.002** | | 0.003 | | 0.003 |
|  | INV | | 0.007 | | 0.008 | | 0.007 | | 0.008 | | 0.008 | | 0.009 | | **0.007** | | 0.009 |
|  | BND | | 0.002 | | 0.002 | | 0.002 | | 0.002 | | 0.002 | | 0.005 | | **0.002** | | 0.005 |
| SvABA | DEL | | 0.025 | | 0.025 | | 0.025 | | **0.025** | | 0.026 | | 0.035 | | 0.026 | | 0.036 |
|  | DUP/INS | | 0.023 | | 0.023 | | 0.023 | | 0.023 | | 0.023 | | 0.028 | | **0.023** | | 0.028 |
|  | INV | | 0.022 | | 0.022 | | 0.022 | | **0.022** | | 0.023 | | 0.033 | | 0.027 | | 0.037 |
|  | BND | | 0.027 | | 0.027 | | 0.027 | | 0.027 | | **0.027** | | 0.034 | | 0.030 | | 0.038 |
| Delly | DEL | | 0.004 | | 0.004 | | 0.004 | | 0.004 | | 0.004 | | 0.004 | | 0.004 | | **0.004** |
|  | DUP | | 0.008 | | 0.008 | | 0.008 | | 0.008 | | **0.008** | | 0.009 | | 0.010 | | 0.010 |
|  | INS | | 0.024 | | 0.024 | | 0.024 | | 0.024 | | 0.024 | | 0.024 | | 0.018 | | **0.017** |
|  | INV | | 0.020 | | 0.020 | | 0.020 | | **0.020** | | 0.021 | | 0.026 | | 0.015 | | 0.023 |
|  | BND | | 0.005 | | 0.005 | | 0.005 | | 0.005 | | 0.005 | | 0.005 | | **0.004** | | 0.005 |
| **F1 score** | | | | | | | | | | | | | | | | | |
| Manta | | DEL | 0.003 | **0.003** | | 0.004 | | 0.004 | | 0.004 | | 0.004 | | 0.005 | | 0.006 | |
|  |  | DUP | 0.006 | 0.006 | | 0.006 | | 0.006 | | **0.006** | | 0.007 | | 0.008 | | 0.009 | |
|  |  | INS | 0.010 | 0.010 | | 0.011 | | 0.011 | | 0.011 | | 0.011 | | **0.011** | | 0.012 | |
|  |  | INV | 0.012 | 0.012 | | 0.012 | | 0.012 | | 0.012 | | 0.013 | | **0.012** | | 0.014 | |
|  |  | BND | 0.004 | 0.004 | | 0.004 | | 0.004 | | 0.004 | | 0.004 | | 0.004 | | **0.004** | |
| Lumpy | | DEL | 0.014 | 0.014 | | 0.014 | | 0.014 | | **0.014** | | 0.017 | | 0.015 | | 0.018 | |
|  |  | DUP | 0.005 | **0.005** | | 0.006 | | 0.006 | | 0.006 | | 0.009 | | 0.006 | | 0.009 | |
|  |  | INV | 0.015 | 0.015 | | 0.015 | | 0.015 | | 0.015 | | 0.024 | | **0.015** | | 0.024 | |
|  |  | BND | 0.009 | 0.009 | | 0.009 | | 0.009 | | **0.009** | | 0.013 | | 0.010 | | 0.013 | |
| GRIDSS | | DEL | 0.010 | 0.010 | | 0.010 | | 0.010 | | 0.010 | | 0.010 | | **0.009** | | 0.010 | |
|  |  | DUP | 0.006 | 0.006 | | 0.006 | | 0.006 | | 0.006 | | 0.006 | | 0.006 | | **0.006** | |
|  |  | INV | 0.006 | 0.006 | | 0.006 | | 0.006 | | 0.006 | | 0.006 | | 0.006 | | **0.006** | |
|  |  | BND | 0.004 | 0.004 | | 0.004 | | 0.004 | | 0.004 | | 0.005 | | **0.004** | | 0.005 | |
| SvABA | | DEL | 0.022 | 0.022 | | 0.022 | | **0.022** | | 0.023 | | 0.035 | | 0.023 | | 0.035 | |
|  |  | DUP/INS | 0.020 | 0.021 | | **0.020** | | 0.021 | | 0.022 | | 0.031 | | 0.021 | | 0.031 | |
|  |  | INV | 0.017 | 0.018 | | **0.017** | | 0.018 | | 0.019 | | 0.028 | | 0.018 | | 0.028 | |
|  |  | BND | 0.018 | 0.019 | | **0.018** | | 0.019 | | 0.020 | | 0.024 | | 0.020 | | 0.024 | |
| Delly | | DEL | 0.012 | 0.012 | | 0.012 | | 0.012 | | 0.012 | | **0.012** | | 0.013 | | 0.030 | |
|  |  | DUP | 0.017 | 0.017 | | 0.017 | | 0.017 | | 0.017 | | **0.017** | | 0.020 | | 0.039 | |
|  |  | INS | 0.014 | 0.014 | | 0.014 | | 0.014 | | 0.014 | | **0.014** | | 0.018 | | 0.044 | |
|  |  | INV | 0.011 | 0.011 | | 0.011 | | **0.011** | | 0.017 | | 0.021 | | 0.013 | | 0.028 | |
|  |  | BND | 0.011 | 0.011 | | 0.011 | | 0.011 | | 0.011 | | **0.011** | | 0.012 | | 0.042 | |
