# Supplementary material for: Shiny-SoSV: A web-based performance calculator for somatic structural variant detection
Source: PLoS One. 2020 Aug 27;15(8):e0238108. doi: 10.1371/journal.pone.0238108 (PMC7451576; doi:10.1371/journal.pone.0238108)
Supplement: S1 File — (DOCX) [file pone.0238108.s021.docx]

**Shiny-SoSV: A web-based performance calculator for somatic structural variant detection**

Tingting Gong^1,2^, Vanessa M Hayes^1,2,3^, Eva KF Chan^1,3*^

^1^Garvan Institute of Medical Research, Darlinghurst, New South Wales, Australia

^2^Central Clinical School, University of Sydney, Camperdown, New South Wales, Australia

^3^St Vincent’s Clinical School, University of New South Wales, Randwick, New South Wales, Australia

**Supplementary material**

**Evaluation of Simulation Design**

To better understand the impact of our simulation design on our prediction models, we examined (1) the impact of different SV sets, (2) stochastic noise of read subsampling, and (3) the impact of the extent of baseline (germline) SVs. To examine the impact of SV simulation, we generated three *in silico* tumour/normal genomes (Set 1, 2 and 3) using three different sets of simulated somatic and germline SVs, with parameter combinations as shown Table 1. To examine the effect of stochastic noise of sequencing read simulation, we replicated SV Set 1 another two times (Sets 1.1, 1.2 and 1.3) by subsampling different paired-end reads from the augmented BAM files. All simulated datasets generated for the purpose here contained an equal number of non-overlapping somatic and germline SVs (1,200 each). This allowed us to compare the data generated here and that reported in the main paper, which we labelled as Set 4 here. Specifically, comparison between Sets 1-3 and Set 4 allowed us to evaluate (a) the difference between 1:1 ratio and 1:10 ratio of somatic to germline SVs and (b) the impact of germline SVs interference by comparing the performance of SV detection when SVs are non-overlapping (as simulated here) and SVs that are possibly overlapping (as simulated for the main paper where SVs were randomly spiked in to the *in silico* genomes).

We found there is little impact from stochastic noise (shown in red, blue and green in Fig 1) and SV positions (purple in Fig 1) associated with SV simulation. However, both the quantity and colocalization of SVs in the simulation (orange in Fig 1) can have a great impact on SV detection.

Increasing germline SVs by 10-fold and distributing SVs randomly (non-overlapping) changes sensitivity and precision by no more than 5% in most cases (85% for sensitivity and 97% for precision) (Fig 2). Among all callers, GRIDSS and Delly have callsets with sensitivity reduced by up to 8% and 15%, with the largest reduction occurring at lower VAF. The largest decrease (> 10%) in sensitivity in Set 4 relative to Sets 1-3, was observed in callsets reported by Delly for SVs present at VAF < 0.5, which could be due to the overall low performance of Delly for low tumour purity samples [10]. The majority (71%) of Manta callsets had sensitivity reduced by 2.5% to 7.5% in Set 4 compared to Sets 1-3. This observation is concordant with previous reports that the depth of coverage of the matched-normal sample generally has larger impact for Manta than other callers examined [10]. Although seemingly large decrease (>10%) in precision were observed in Set 4 compared to Sets 1-3, most were artefacts due to small total number of reported SVs in both Sets 1-3 and Set 4, particularly at low tumour coverage (20x), low VAF (0.05) and stringent breakpoint precision threshold (2bp). For example, SvABA only reported one SV for three scenarios (tumour coverage of 20x, VAF of 0.05 and breakpoint threshold of 2bp) in Set 4, which directly impacts the number of total true positives, thus resulting in 100% increase in precision.

Table 1. Simulated and evaluated variables for Set1-3

| Set1 | | | |  | Set2 | | | |  | Set3 | | | |
| --- | --- | --- | --- | --- | --- | --- | --- | --- | --- | --- | --- | --- | --- |
| Tumour coverage | Normal coverage | VAF | *T* (bp)^1^ |  | Tumour coverage | Normal coverage | VAF | *T* (bp)^1^ |  | Tumour coverage | Normal coverage | VAF | *T* (bp)^1^ |
| 20x  30x  45x  60x  75x  90x | 15x  30x  45x  60x  75x  90x | 0.05  0.1  0.2  0.5  0.8  1 | 2  5  50  100  150  200 |  | 25x  38x  50x  70x  85x  95x | 25x  38x  50x  70x  85x  95x | 0.12  0.25  0.4  0.6  0.75  0.95 | 8  15  30  90  120  180 |  | 20x  35x  40x  60x  80x  100x | 15x  35x  40x  60x  80x  100x | 0.05  0.15  0.3  0.5  0.7  0.9 | 2  10  60  100  130  190 |

^1^*T* is breakpoint precision threshold. This is the maximum allowed difference between called and simulated breakpoint positions to be defined as true positive.


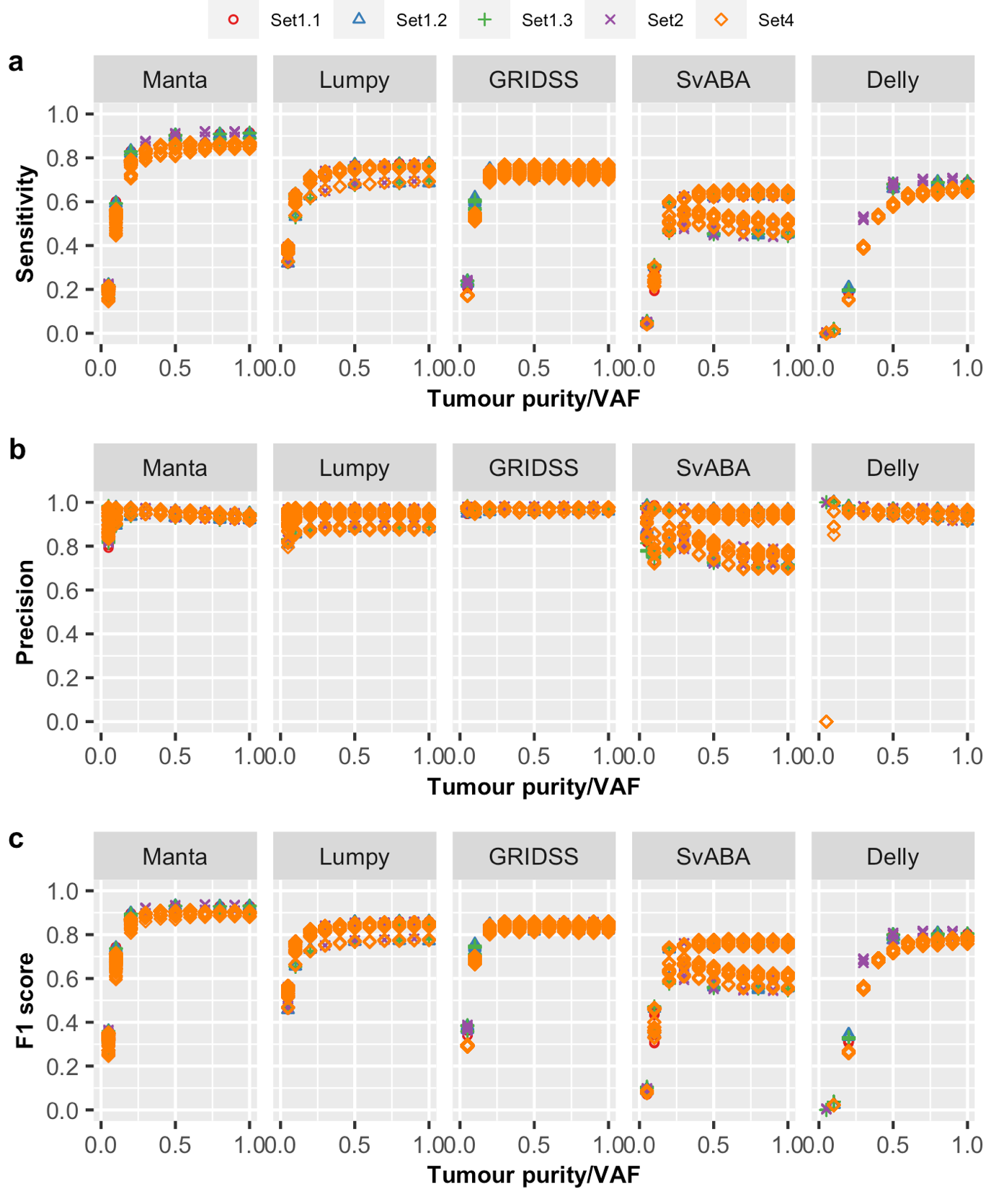


**Fig 1.** **Evaluation results comparison for different SV simulation design.** Shown are the (a) sensitivity, (b) precision and (c) F1 score across tumour purity/VAF for five SV callers (Manta, Lumpy, GRIDSS, SvABA, Delly), evaluated on three replicates of simulation SV Set 1 (Set1.1, Set1.2, Set1.3), 2 and 3. Simulation Set 1 and 2 have same number of non-overlapping 1,200 somatic and germline SVs, while Set3 has randomly placed 12,000 germline SVs. Results shown are based on variables with breakpoint precision threshold of 2bp, 10bp, 60bp, 100bp and 200bp, normal coverage of 30x, 40x, 60x and 90x and tumour coverage of 60x.


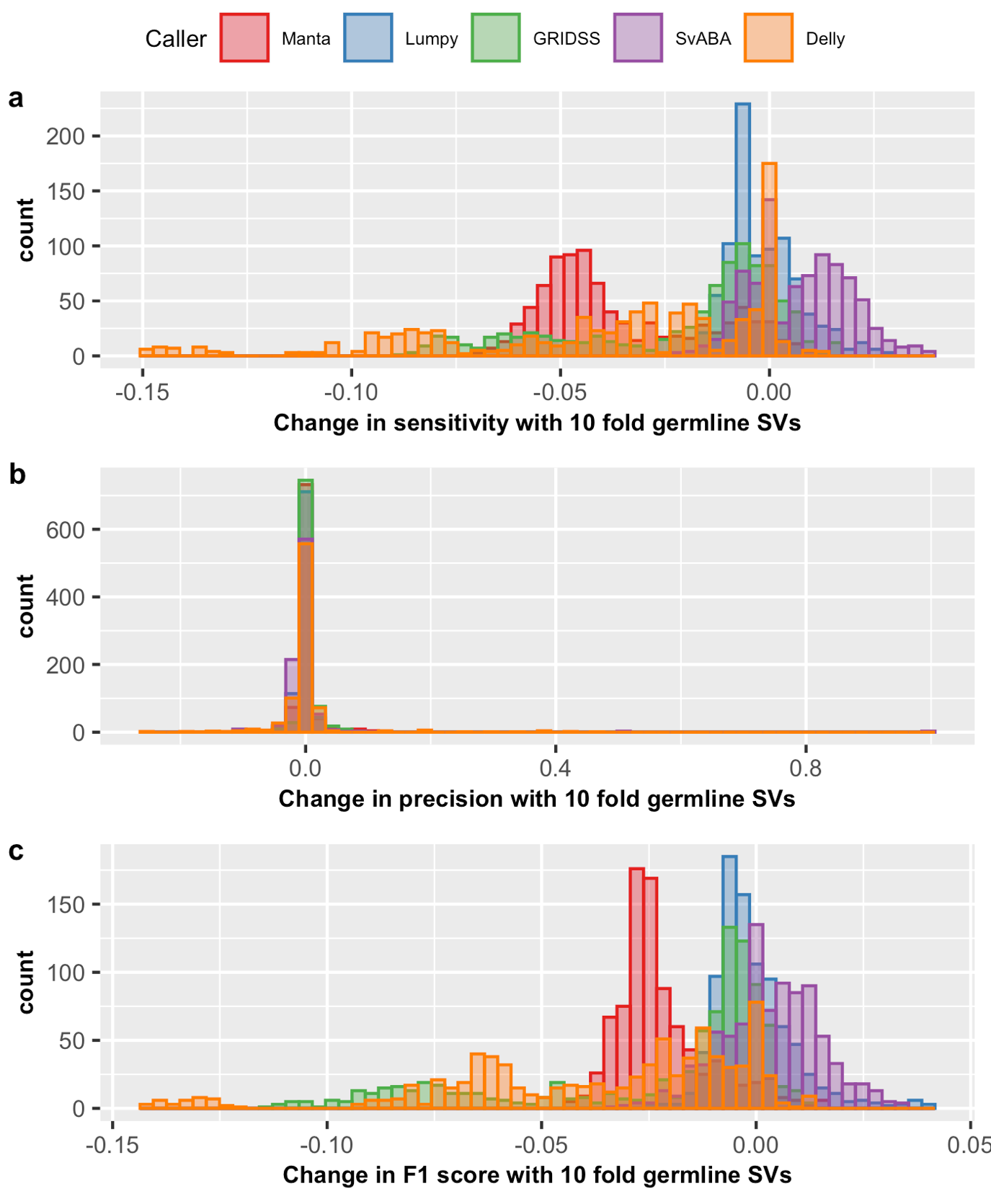


**Fig 2.** **The spread of change in evaluation results.** Shown are the frequency of change in (a) sensitivity, (b) precision, comparing simulation Set 4 to other three simulation sets for five SV callers (Manta, Lumpy, GRIDSS, SvABA, Delly).

**Evaluation and predictive model selection of SV type detection**

As previously reported, there is variability in detection performance for different SV types, where in general DEL has the best performance among all SV types with any combinations of predictive variables, while INS is hard to detect [10].

Specifically, detection sensitivity was lowest for FINS [10]. This is because SV detection relies on alignment signatures of sequencing reads, and in the case of FINS, where the inserted sequence is absent from the reference genome, these alignment signatures are substantially weakened or completely absent in some cases. Manta has the highest sensitivity for FINS detection because it reports large INS even though the inserted sequence cannot be fully assembled. Due to the limitations of FINS detection, the positive effect of VAF and tumour coverage on sensitivity were much less obvious for FINS than other SV types (**S5-8** **Fig**). As no FINS was detected by Lumpy and SvABA, this SV type was not included for further sensitivity predictive model selection and fitting for these two callers.

Precision and F1 score values of each SV type are shown in **S9-12** **Fig** and **S13-16** **Fig** respectively. We note that some combinations of predictors result in very few positive calls, which can significantly inflate precision estimates. For example, only 2 DUP were called by Manta at tumour and normal depth of coverage of 20x and VAF of 0.05, resulting in 100% precision. Such low number of datapoints may result in artefactual precision evaluation and imprecise prediction. In particular, few positive calls (<30) were reported at low VAF (VAF <= 0.1) and low tumour coverages (<=20x), and also for INS calls by GRIDSS. As such, these callsets were not included in predictive model selection and fitting for precision and F1 score values.

Given performance differences for different SV types, we also evaluated all eight prediction models (Model (1) – (8)) independently for each SV type. The RMSE for each candidate model and each SV type for five SV callers are reported in **S4 Table**. The GAM model achieving the lowest error rate for each SV type was selected. Interestingly, despite detection variability between SV types, the impact of predictor variables on the detection of individual SV type (**S5-16 Fig**) is similar to the impact on overall performance (**Fig 1-3**).
